# Ruptured aortic aneurysm with previous endovascular aneurysm repair in a patient with high-grade angiosarcoma

**DOI:** 10.1016/j.jvscit.2024.101610

**Published:** 2024-08-22

**Authors:** Ievgen Gegiia, Félix H. Savoie-White, Anthony Calabrino, Violaine Dalens, Pascal Rhéaume, Annie Boisvert

**Affiliations:** a Division of Vascular Surgery, CHU de Québec, Québec, Canada; b Division of Internal Medicine, CHU de Québec, Québec, Canada

**Keywords:** Aortic aneurysm rupture, Angiosarcoma, Endovascular treatment

## Abstract

Ruptured aortic aneurysms after endovascular repair is rare, particularly in the absence of type I or type III endoleaks. In such cases, a thorough investigation into the causes is imperative, including the consideration of an underlying malignancy. We report a case involving a 78-year-old woman who experienced abdominal aortic aneurysm rupture 4 years after aortic endograft treatment. We explanted the endograft and performed aortobi-iliac bypass. Initial aortic thrombus pathological analysis revealed atherosclerosis. However, the patient returned 4 months later with multiple lesions suggestive of metastases, and a reevaluation of the pathology slides uncovered a diagnosis of angiosarcoma.

A ruptured aortic aneurysm after endovascular repair without evident unstable endoleaks is unusual and warrants an investigation for potential causes such as infection or cancer.^1^^,^^2^ Angiosarcoma is a rare type of cancer that has been described in patients after endovascular aortic repair and remains a challenging diagnosis.^3^ We describe the case of a 78-year-old patient who suffered a ruptured abdominal aortic aneurysm 4 years after endograft placement. Surgical intervention included endograft explantation and open bypass surgery. Initial aortic thrombus pathology suggested atherosclerosis. Subsequent readmission and pathology review four months later revealed angiosarcoma. This case report has been reported in line with the Surgical CAse REport criteria.^4^

Written publication consent was received from the patient.

## Case report

**Fig 1:**
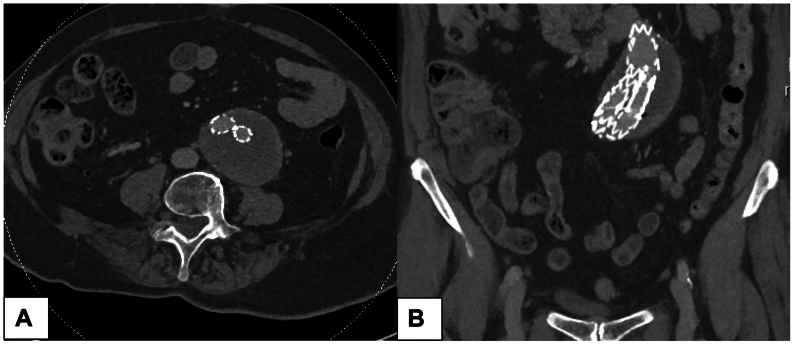
Initial computed tomography (CT) angiogram showing axial **(A)** and coronal **(B)** views on first follow-up CT scan after endovascular aneurysm repair (EVAR).

**Fig 2:**
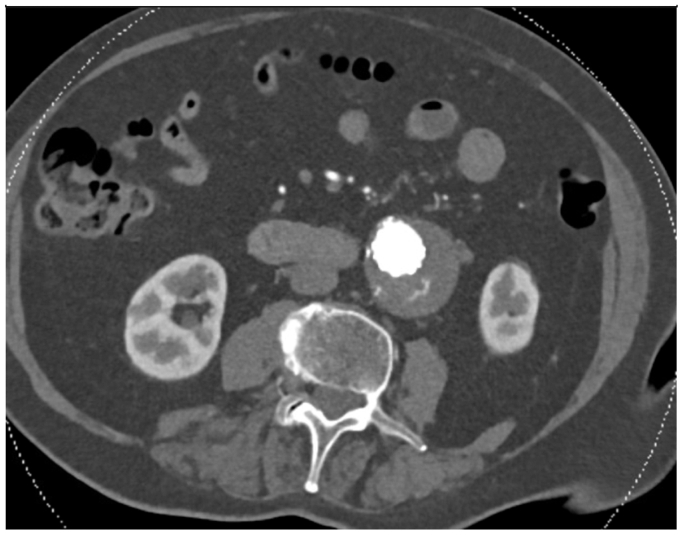
Computed tomography (CT) angiogram showing axial view of type II endoleak after endovascular aneurysm repair (EVAR).

A 78-year-old woman with a history of breast cancer in remission, hypertension, dyslipidemia, and coronary artery disease presented to the emergency department with acute abdominal pain radiating to the back, nausea, and vomiting. She did not present fever nor weight loss. Her past surgical history was significant for an infrarenal abdominal aortic aneurysm that was repaired 4 years prior with an Endurant II Stent Graft System (Medtronic, Dublin, Ireland) (Fig 1). Four years after repair, imaging revealed a type II endoleak (Fig 2) that was surveilled with annual computed tomography (CT) angiogram. A CT scan performed 9 months before presentation showed a stable aneurysmal sac, with a diameter similar to the aneurysm at the time of endovascular aneurysm repair (EVAR) implantation.

**Fig 3:**
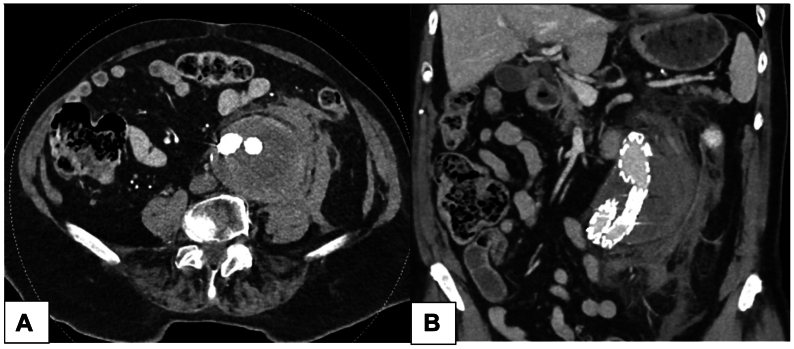
Computed tomography (CT) angiogram showing axial **(A)** and coronal **(B)** views of contained abdominal aortic aneurysm (AAA) rupture after endovascular aneurysm repair (EVAR).

Upon admission, the patient was hemodynamically stable. Laboratory tests revealed leukocytosis (18.8 × 10^9^/L) with a left shift, normal hemoglobin at 12.1 g/dL, and mild acute renal failure (creatinine of 1.091 mg/dL). CT findings (Fig 3) demonstrated a periaortic hematoma and a progression of the aneurysm sac size to 6.9 cm compared with 5.7 cm 9 months prior. The known type II endoleak was still present, but there was no contrast extravasation outside the aneurysm, and the rupture site could not be identified. The imaging was consistent with a contained rupture of the abdominal aortic aneurysm.

The patient underwent a partial explantation of her EVAR via a transperitoneal approach with an infrarenal clamp site. As there was no suspicion of infection, the main body was severed at the level of the infra-renal neck, leaving the sealing zone portion in situ. An aortobi-iliac bypass (Gelsoft Plus) was performed, with the ligation of a lumbar artery for hemostasis. The total blood loss was 2.9 L, and the operative time was 2 hours and 25 minutes. The postoperative course was uneventful in terms of surgical complications; however, the patient developed an hospital-acquired pneumonia and delirium. She was discharged from the intensive care unit on postoperative day 5 and from the hospital on postoperative day 24. As per our protocol, the explanted endograft, aortic wall tissue, and thrombus were sent for culture and routine pathological examination, which initially revealed atherosclerosis. Blood cultures taken on postoperative day 1 were negative.

The patient was readmitted to the hospital 4 months later with worsening symptoms of fatigue, difficulty breathing, and frequent falls. Vital signs were normal, but lab results showed low hemoglobin (65 g/L). Blood and urine cultures were negative. CT scans showed a mild decrease in the periaortic infiltration, without signs of infection. Imaging also revealed bilateral pulmonary opacities and pleural effusion with some hemorrhage. Thoracenteses yielded hemosiderin-laden macrophages. The transthoracic and transesophageal echocardiograms showed normal ventricular function without significant valve anomaly or ventricular/atrial thrombus.

**Fig 4:**
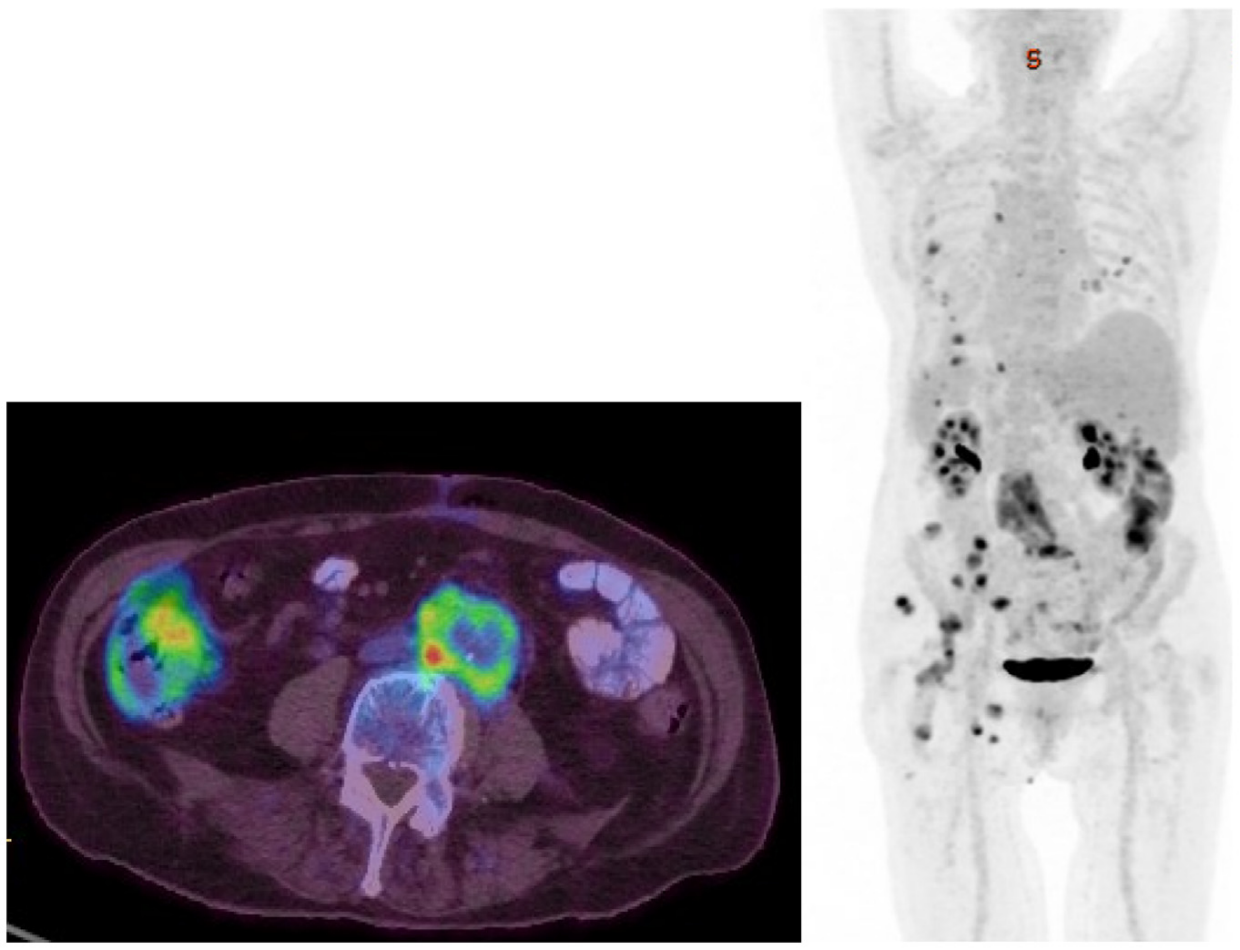
Positron emission tomography scan demonstrating intense hypermetabolic area surrounding aortobi-iliac bypass after endovascular aneurysm repair (EVAR) explantation.

**Fig 5:**
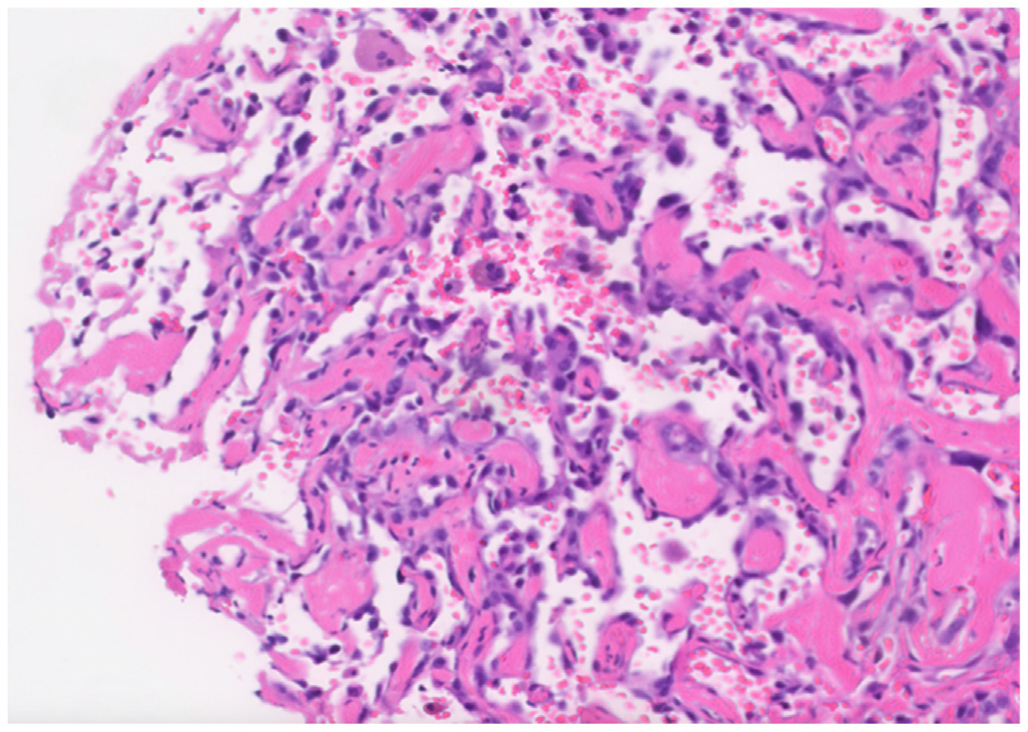
Pathology slide showing atypical endothelial cells infiltrating in the aortic thrombus consistent with angiosarcoma.

A positron emission tomography scan was performed (Fig 4), and findings were notable for hypermetabolism surrounding the aortobi-iliac bypass as well as multiple other hypermetabolic sites, including bilateral pulmonary parenchyma, left lung pleura, and soft tissue structures such as subcutaneous tissue at the level of the left buttock, left femur diaphysis, left ilium, and sacrum. The working differential diagnosis included graft infection, septic emboli, and metastatic malignancy. The patient was empirically treated with broad-spectrum antibiotics. Gallium scintigraphy showed diffuse activity at the left talus and calcaneum and a CT-guided biopsy was performed. The pathology report revealed a high-grade angiosarcoma (Fig 5). Given the new diagnosis of neoplasia, a reanalysis of the aortic thrombus pathology from the explantation was requested. Upon reviewing the slides, the pathologist identified atypical vascular proliferation within the surrounding cells from the original aortic thrombus consistent with a portion of angiosarcoma (Fig 5).

A follow-up positron emission tomography scan revealed increased pulmonary and pleural hypermetabolism, as well as advancing skeletal involvement. Treatment recommendations, including chemotherapy, were presented to the patient who declined treatments and preferred palliative care.

## Discussion

Angiosarcoma is a rare type of cancer, representing approximately 2% of all soft tissue sarcomas.^5^ Angiosarcoma presents a diagnostic challenge owing to its vague symptoms, complicating early detection and exacerbating its aggressive nature. It is particularly problematic in patients with aortic grafts, because it is often misdiagnosed as a graft-related complication. The diagnosis typically happens late, often with metastases, and after tumor resection or on postmortem examinations.

The efficacy of adjuvant chemotherapy and/or radiotherapy, either alone or in conjunction with surgical resection, remains a contentious topic in oncology. Despite these treatments, survival rates are alarmingly low. These cancers exhibit a 5-year survival rate of approximately 35%, with a median survival time of merely 7 months, and a mere 4% survival rate beyond 10 years.^6^^,^^7^

In our case, the patient experienced rapid aneurysm sac expansion leading to rupture, initially without a clear cause. Standard CT angiography revealed no evidence of infection or malignancy. The initial pathology analysis indicated atherosclerosis as the main diagnosis. However, post-EVAR angiosarcomas should be included in the differential diagnosis.^8^, ^9^, ^10^, ^11^ In the literature, similar cases showed symptoms of distal embolization or signs of infection. In some instances, imaging revealed tumors spreading to the lumbar vertebrae and retroperitoneal lymph. If concomitant post-EVAR infection is suspected, a rifampin-soaked graft should be used after endograft explantation.^12^

The rarity of angiosarcoma after EVAR highlights the importance for clinicians to approach unusual clinical presentations with a comprehensive mindset. Rapidly expanding aneurysm sacs on CT scans, particularly without signs of infection or endoleak, should prompt careful consideration. Although the initial inclination may lean toward common post-EVAR complications, the presence of such a rare malignancy, as seen in our case, stresses the need for clinicians to maintain a heightened suspicion.

Additionally, differences in institutional practices, such as whether aortic thrombus samples are sent for pathological evaluation, may lead to missed opportunities for early detection of malignancy. This raises questions about standardizing post-EVAR management and the significance of establishing uniform best practices across institutions. A multidisciplinary approach involving radiologists, internists, pathologists, infectious disease specialists, and vascular surgeons can enhance diagnostic accuracy and ensure optimal patient care.

## Conclusions

Aortic rupture after EVAR is an infrequent but significant event. Immediate recognition and thorough investigation to determine the underlying cause are crucial. In such circumstances, it is essential to consider malignancies like angiosarcoma in the differential diagnosis due to their association with a dismal prognosis and diagnostic challenges.

## Disclosures

None.
